# Infectious salmon anaemia virus replication and induction of alpha interferon in Atlantic salmon erythrocytes

**DOI:** 10.1186/1743-422X-5-36

**Published:** 2008-02-28

**Authors:** Samuel T Workenhe, Molly JT Kibenge, Glenda M Wright, Dorota W Wadowska, David B Groman, Frederick SB Kibenge

**Affiliations:** 1 Department of Pathology and Microbiology, Atlantic Veterinary College, University of Prince Edward Island, 550 University Avenue, Charlottetown, PE. C1A 4P3, Canada; 2 Department of Biomedical Sciences, Atlantic Veterinary College, University of Prince Edward Island, 550 University Avenue, Charlottetown, PE. C1A 4P3, Canada; 3 Electron Microscopy Laboratory, Atlantic Veterinary College, University of Prince Edward Island, 550 University Avenue, Charlottetown, PE. C1A 4P3, Canada; 4 Aquatic Diagnostic Services, Atlantic Veterinary College, University of Prince Edward Island, 550 University Avenue, Charlottetown, PE. C1A 4P3., Canada

## Abstract

**Background:**

Infectious salmon anaemia (ISA) virus (ISAV), which causes ISA in marine-farmed Atlantic salmon, is an orthomyxovirus belonging to the genus *Isavirus*, family *Orthomyxoviridae*. ISAV agglutinates erythrocytes of several fish species and it is generally accepted that the ISAV receptor destroying enzyme dissolves this haemagglutination except for Atlantic salmon erythrocytes. Recent work indicates that ISAV isolates that are able to elute from Atlantic salmon erythrocytes cause low mortality in challenge experiments using Atlantic salmon. Previous work on ISAV-induced haemagglutination using the highly pathogenic ISAV strain NBISA01 and the low pathogenic ISAV strain RPC/NB-04-0851, showed endocytosis of NBISA01 but not RPC/NB-04-0851. Real-time RT-PCR was used to assess the viral RNA levels in the ISAV-induced haemagglutination reaction samples, and we observed a slight increase in viral RNA transcripts by 36 hours in the haemagglutination reaction with NBISA01 virus when the experiment was terminated. However, a longer sampling interval was considered necessary to confirm ISAV replication in fish erythrocytes and to determine if the infected cells mounted any innate immune response. This study examined the possible ISAV replication and Type I interferon (IFN) system gene induction in Atlantic salmon erythrocytes following ISAV haemagglutination.

**Results:**

Haemagglutination assays were performed using Atlantic salmon erythrocytes and one haemagglutination unit of the two ISAV strains, NBISA01 and RPC/NB-04-0851, of differing genotypes and pathogenicities. Haemagglutination induced by the highly pathogenic NBISA01 but not the low pathogenic RPC/NB-04-0851 resulted in productive infection as evidenced by increased ISAV segment 8 transcripts and increase in the median tissue culture infectious dose (TCID_50_) by 5 days of incubation. Moreover, reverse transcription (RT) quantitative PCR used to compare mRNA levels of key Type I IFN system genes in erythrocyte lysates of haemagglutination reactions with the two ISAV strains showed a higher relative fold increase of IFN-α in NBISA01 haemagglutinations compared to RPC/NB-04-085-1 haemagglutinations (33.0 – 44.26 relative fold increase compared to 11.29). Erythrocytes exposed to heat-inactivated virus or to polyinosinic:polycytidylic acid (polyI:C) or to L-15 medium alone (negative control assays) had minimal late induction (<3.5 relative fold increase) of STAT1 and/or ISG15 and Mx genes, whereas erythrocytes exposed to UV-inactivated virus lacked any cytokine induction.

**Conclusion:**

ISAV-induced haemagglutination by a highly pathogenic virus strain results in virus uptake and productive infection of Atlantic salmon erythrocytes accompanied by significant induction of IFN-α. This study also highlights the critical role of ISAV strain variation in the initial stages of the virus-cell interaction during haemagglutination, and possibly in the pathogenesis of ISA. Moreover, the study shows for the first time that fish erythrocytes immunologically respond to ISAV infection.

## Background

Infectious salmon anaemia (ISA) is a highly fatal viral disease affecting marine-farmed Atlantic salmon (*Salmo salar *L). This fish disease is caused by ISA virus (ISAV), a fish orthomyxovirus assigned to the genus *Isavirus *within the family *Orthomyxoviridae *[1]. The ISAV strains are enveloped particles of 90–140 nm diameter with surface projections consisting of a combined haemagglutinin-esterase (HE) protein [2] and a separate fusion (F) protein [3]. The genome is composed of eight segments of linear, single-stranded negative sense RNA ranging in length from 1.0 to 2.4 kb with a total molecular size of approximately 14.3 kb [4].

The clinical disease caused by ISAV in marine-farmed Atlantic salmon is associated with anaemia [5], which is hypothesized to be linked with uptake of virus-coated erythrocytes by immune cells [6]. The fish erythrocytes would probably be coated with ISAV through interaction of the cellular sialic acid receptors and the viral HE glycoprotein as occurs during the haemagglutination reaction. ISAV haemagglutination of fish erythrocytes, similar to influenza A virus haemagglutination of avian and mammalian erythrocytes, involves three independent phenomena: [1] adsorption of viruses at the erythrocyte membrane, [2] subsequent elution [7-9], which is not always complete, and [3] uptake of viruses by the erythrocytes [10,11]. For ISAV, elution from erythrocytes was originally reported to occur with erythrocytes of several fish species except Atlantic salmon [7] in which the virus causes a natural clinical disease (as reviewed in [12]). However, recent work indicates that ISAV isolates that are able to elute from Atlantic salmon erythrocytes cause low mortality in challenge experiments using Atlantic salmon [13]. In our previous work on ISAV-induced haemagglutination using the highly pathogenic NBISA01 and the low pathogenic RPC/NB 04-0851, only NBISA01 was taken up by the erythrocytes from Atlantic salmon and rainbow trout (*Oncorhynchus mykiss*) [11]. In contrast, the uptake of influenza A virus by avian and mammalian erythrocytes via pinocytosis was non-specific [10] indicating a lack of involvement of virus strain-specific differences such as pathogenicity level. This suggested to us that the lack of dissolution of pathogenic ISAV-induced haemagglutination of Atlantic salmon erythrocytes favours endocytosis of the virus particles by the erythrocytes [11] and this phenomenon may contribute to the anaemia in ISA.

Members of the family *Orthomyxoviridae *are known to have a nuclear replication phase [1]. Enucleated BS-C-1 cells have been shown to be non-permissive for replication of influenza A viruses [14]. By inference, mature mammalian erythrocytes, which are enucleated, are also non-permissive for replication of influenza A viruses. In the case of the nucleated avian (turkey or chicken) erythrocytes, haemagglutination by avian influenza A virus resulted only in *de novo *synthesis of viral proteins but not production of new infectious virus [15]. We previously used real-time RT-PCR to assess the viral RNA levels in the ISAV-induced haemagglutination reaction samples, and observed a slight increase in viral RNA transcripts by 36 hours in the haemagglutination reaction with NBISA01 virus when the experiment was terminated [11]. However, a longer sampling interval was considered necessary to confirm ISAV replication in fish erythrocytes and to determine if the infected cells mounted any innate immune response.

The Type I IFN system constitutes the major antiviral defence mechanism in the innate immune system of mammals as well as fish [16]. Most cell types are able to detect viral replication dsRNA intermediates and respond by secretion of IFN, which then uses the JAK/STAT signalling pathway to stimulate induction of the components of the Type I IFN system genes such as Mx, ISG15 and STAT1 [17]. Atlantic salmon organs and the macrophage-like fish cell line TO [18] respond to ISAV infection by up-regulating the expression of key Type I IFN system genes [19]. The limited immunological studies on the nucleated fish erythrocytes suggest that they possess a certain level of immune functions; the mature erythrocyte populations of rainbow trout were shown to surround macrophages phagocytosing *Candida albicans *and to secrete cytokine-like macrophage inhibitory factors [20]. However, there is no report of IFN induction in intact erythrocytes either in fish or avian or mammalian species.

The goal of this study was to determine whether ISAV uptake by fish erythrocytes results in a productive infection and, if so, whether there is any effect of differences in the pathogenicity level of the virus on the cellular response. This information is needed to further clarify the pathogenesis of the clinical disease in fish. In order to obtain information on the putative associated innate immune response in erythrocytes, we used reverse transcription (RT) quantitative PCR assays with SYBR Green chemistry to evaluate mRNA levels of Type I IFN system genes IFN-α, Mx, ISG15, STAT1 and PKZ at regular intervals up to 5 days following virus-induced haemagglutination.

## Results

### ISAV replicates in Atlantic salmon erythrocytes

Haemagglutination by pathogenic ISAV is associated with endocytosis of the virus particles, which seems to be consistent with virus infection [11]. To determine whether ISAV endocytosis by fish erythrocytes results in a productive infection, and to further analyze the differences between virus strains of differing pathogenicity, we monitored the haemagglutination assays with NBISA01 and RPC/NB-04-085-1 strains for transcription of viral genes on ISAV segment 8 using real-time RT-PCR. In the previous report, the haemagglutination assays were carried out with 10^9.75 ^TCID_50_/ml for NBISA01 and 10^4.25 ^TCID_50_/ml for RPC/NB-04-085-1 [11]. In order to use an equal number of haemagglutination (HA) units in the present study, the HA units of the stock virus preparations were determined, and all haemagglutination assays used 1 HA unit of virus preparation. For NBISA01, 1 HA unit contained 10^8.45 ^TCID_50 _and had a cycle threshold (Ct) value of 26.57, whereas for RPC/NB-04-085-1, 1 HA unit contained 10^5.75 ^TCID_50 _and had a Ct value of 20.39. Thus, the present study, by using the standard 1 HA unit in haemagglutination assays, had less NBISA01 virus but more RPC/NB-04-085-1 virus content in each haemagglutination reaction than in our previous study [11]. It was possible to maintain the erythrocytes viable for up to 5 days in haemagglutination assays in the present study by changing the medium of the haemagglutination assay from PBS to L-15 growth medium. The real-time RT-PCR data for quantification of viral transcripts are presented in Table 1. All the Ct values were confirmed to be for positive amplicons by melting curve analysis. Agarose gel electrophoresis of the RT-PCR products also confirmed virus-specific amplification for NBISA01 and RPC/NB-04-085-1, whereas Atlantic salmon erythrocytes without virus, which were incubated in L-15 medium alongside the haemagglutinations as a negative control showed no virus-specific amplification by both melting curve analysis and agarose gel electrophoresis (data not shown). The Ct values for the highly pathogenic NBISA01 strain show a steady decline from the 0 hour (26.57 ± 0.14) to day 5 (20.48 ± 0.29) indicating an increase in viral gene transcription. To confirm if the decrease in Ct value was from newly synthesized viral mRNA we used oligodT primers for cDNA synthesis followed by real-time PCR amplification. A similar decrease in Ct value from 0 hour (22.22 ± 0.05) to day 5 (19.29 ± 0.12) was observed. In this case, the Ct value of 22.22 at time 0, at which no viral mRNA should be present, was attributed to residual transcripts in the viral inocula which were cell culture lysates. For a PCR reaction with 100% efficiency, a 3.3 Ct difference between two samples is equal to a 10-fold difference in starting sample concentration [21]. The F5/R5 primer pair used in the present study has an amplification efficiency of 96.842%. Thus the changes in the Ct values for NBISA01 at each sampling point beginning at day 2 (for the one-step RT-PCR using F5/R5) or day 3 (for two-step RT-PCR with RT primed with oligodT and PCR primed using F5/R5) corresponded to more than 10-fold increase in amplicons in the starting sample concentrations from that of the 0 hour, suggesting that there was *de novo *synthesis of viral RNA in the erythrocytes of Atlantic salmon. In contrast, the low pathogenic RPC/NB-04-085-1 strain showed no significant change in the Ct values for any time point (Table 1), indicating absence of virus replication.

**Table 1:** Transcript levels of viral genes on ISAV segment 8 in extended haemagglutination assays^1^

Sampling points in days	NBISA01 haemagglutination		RPC/NB-04-085-1 haemagglutination
	
	One-step RT-PCR with F5/R5 primer pair	RT with oligodT primer & PCR with F5/R5 primer pair	One-step RT-PCR with F5/R5 primer pair
0	26.57 ± 0.14	22.22 ± 0.05	20.39 ± 0.21
1	24.43 ± 0.15	20.63 ± 0.05	21.23 ± 0.15
2	22.25 ± 0.21*	19.40 ± 0.02	20.46 ± 0.26
3	21.51 ± 0.33*	18.31 ± 0.02*	20.76 ± 0.36
4	21.04 ± 0.40*	18.67 ± 0.27*	20.27 ± 0.22
5	20.48 ± 0.29*	19.29 ± 0.12*	20.55 ± 0.29

^1^Ct values are average ± SD of triplicate observation.

*denotes more than 3.3 Ct difference in the starting sample concentrations from that of the 0 hour.

We examined if the *de novo *synthesis of viral RNA by NBISA01 in the erythrocytes of Atlantic salmon was accompanied with production of new infectious virus by titrating the haemagglutination reactions on the TO cell line, which is highly permissive for ISAV [18,22]. For this, the haemagglutination reactions were sampled at 0, 3 and 5 days post-haemagglutination and 10-fold dilutions of each sample were inoculated on TO cell monolayers in quadruplicate. The 0 hour sample showed a TCID_50 _of 10^7.0 ± 0.433^/ml and day 3 sample showed a TCID_50 _of 10^6.92 ± 0.143^/ml while the day 5 sample had a TCID_50 _of 10^7.75 ± 0.25^/ml, demonstrating a 0.75 log_10 _increase in virus titre by day 5. This indicated a productive infection during ISAV-induced haemagglutination with the highly pathogenic NBISA01 virus. The significant decrease in Ct value in contrast to the small increase in virus titre is attributed to differences in sensitivity between the two assays used here to demonstrate virus replication, and to the limited viral replication possible during the haemagglutination reaction.

### ISAV-induced haemagglutination induces IFN-α in fish erythrocytes

**Figure 1:**
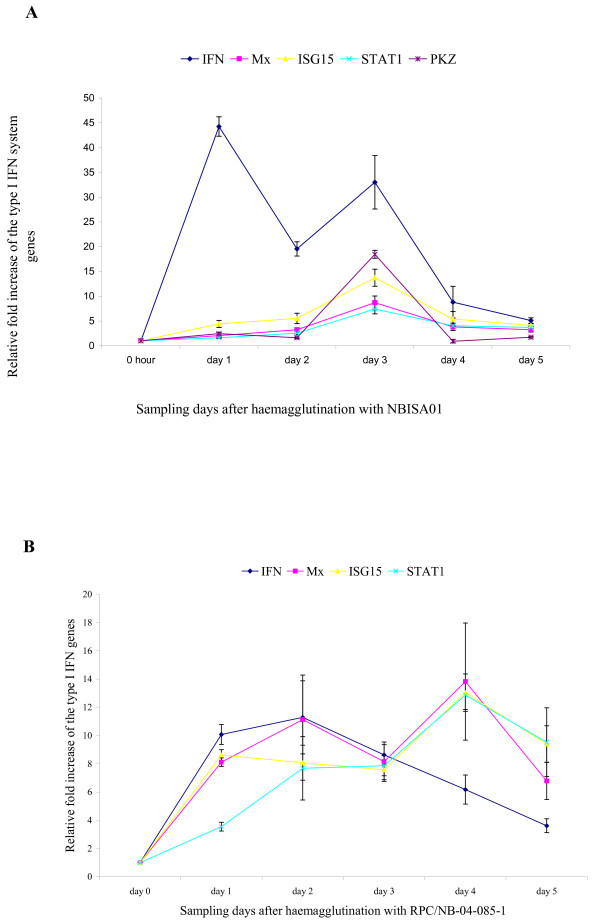
**mRNA levels of key Type I IFN system genes in Atlantic salmon erythrocytes in response to native virus during haemagglutination assay**. Relative fold increase of the key Type I IFN system genes in response to NBISA01 or RPC/NB-04-085-1 haemagglutination calibrated to the 18S rRNA housekeeping gene and the 0 hour control (values are average of a triplicate observation ± standard deviation): **(A) **relative fold stimulation of Atlantic salmon IFN genes (SasaIFN-α1 and SasaIFN-α2), Mx, ISG15 and STAT1 by NBISA01 virus; **(B) **relative fold stimulation of Atlantic salmon IFN genes (SasaIFN-α1 and SasaIFN-α2), Mx, ISG15 and STAT1 by RPC/NB-04-085-1.

**Figure 2:**
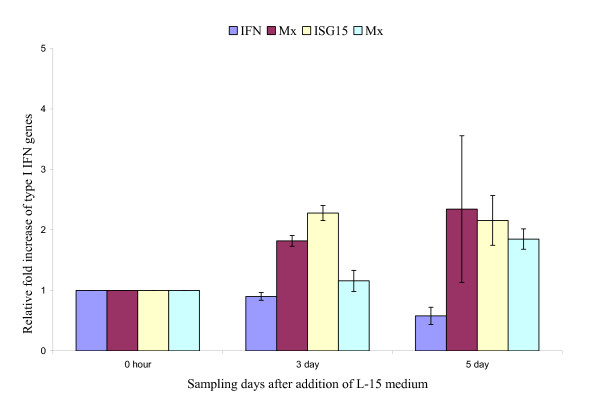
**mRNA levels of key Type I IFN system genes in Atlantic salmon erythrocytes in L-15 medium (negative control for haemagglutination assay)**. Relative fold stimulation of the key Type I IFN system genes in Atlantic salmon erythrocytes when virus is not present; i.e., in response to L-15 medium alone (negative control) calibrated to the 18S rRNA housekeeping gene and the 0 hour control (values are average of a triplicate observation ± standard deviation): Atlantic salmon IFN genes (SasaIFN-α1 and SasaIFN-α2), Mx, ISG15 and STAT1 in negative control erythrocytes.

**Figure 3:**
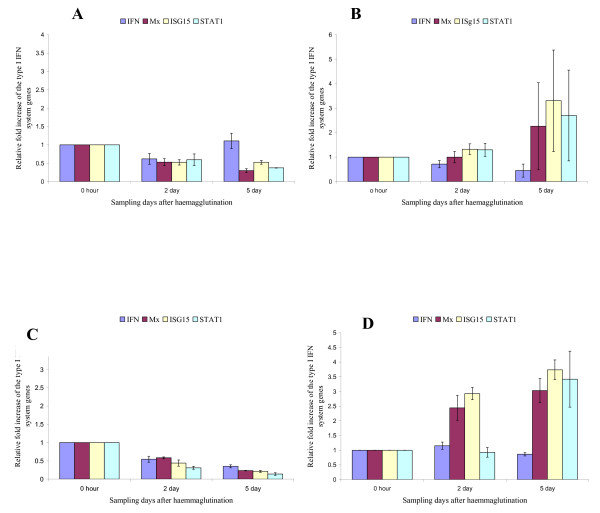
**mRNA levels of key Type I IFN system genes in Atlantic salmon erythrocytes in response to inactivated virus during haemagglutination assay**. Relative fold increase of the key Type I IFN system genes calibrated to the 18S rRNA housekeeping gene and the 0 hour control (values are average of a triplicate observation ± standard deviation): **(A) **UV-inactivated NBISA01; **(B) **heat-inactivated NBISA01; **(C) **UV-inactivated RPC/NB-04-085-1; and **(D) **heat inactivated RPC/NB-04-085-1 haemagglutination.

It is generally accepted that key proteins of the Type I IFN system are induced during ISAV infection but they are unable to inhibit the replication of ISAV *in vitro *and *in vivo *[19]. Constitutive expression in CHSE-214 cells of Atlantic salmon IFN-induced Mx1 protein does, however, confer resistance to ISAV [23]. Moreover, recently, the ISAV segment 7 ORF1 product was reported to be an IFN-signalling antagonist that enables the virus to counteract IFN-induced antiviral proteins of the host, a function similar to that of the non-structural (NS1) protein encoded by segment 8 of influenza A virus [24]. To determine whether fish erythrocytes mount any cytokine response to ISAV during haemagglutination, and to show the effect of virus replication on the quality of the response, we used quantitative RT-PCR assays to evaluate mRNA levels of key Type I IFN system genes IFN-α, Mx, ISG15, STAT1, and PKZ at regular intervals during haemagglutination reactions using native virus and virus inactivated by exposure either to UV light or to heat. The data are summarized in Figures 1 and 2. The data show that the highly pathogenic NBISA01 virus had a higher relative fold increase for IFN-α transcripts than the less pathogenic RPC/NB-04-085-1 virus that did not replicate in erythrocytes. NBISA01 haemagglutinations showed increased IFN-α transcript levels, with a biphasic peak at day 1 (44.26 ± 1.95) and day 3 (33.0 ± 5.4) (Fig. 1A). In contrast, the RPC/NB-04-085-1 haemagglutinations showed a moderate increase by day 2 (11.29 ± 2.59) (Fig. 1B). NBISA01 induced haemagglutinations had a statistically significant (p < 0.05) mean fold increase compared to RPC/NB-04-085-1 haemagglutinations at all sampling days except day 4. The Mx transcript levels in the NBISA01 haemagglutinations were moderate with a maximum by day 3 (8.71 ± 1.33) (Fig. 1A). Surprisingly the Mx transcript levels in the RPC/NB-04-085-1 haemagglutinations had a statistically significant (p < 0.05) mean fold increase compared to NBISA01 haemagglutinations at all sampling days except day 3. ISG15 transcripts had a similar maximum peak for erythrocytes haemagglutinated with either NBISA01 or RPC/NB-04-085-1, except that the peak was by day 3 for NBISA01 where there was a statistically significant mean fold increase compared to RPC/NB-04-085-1. For RPC/NB-04-085-1, days 1, 4, and 5 showed statistically significant mean fold increases of ISG15 compared to NBISA01. STAT1 is a signal transducer and activator of transcription involved in the JAK/STAT signalling for IFN response (reviewed in [17]). NBISA01 haemagglutinations showed up-regulation of STAT1 by day 3 (7.42 ± 0.98). In contrast, RPC/NB-04-085-1 haemagglutinations showed a more stable up-regulation from day 2 to day 5 (Figs 1A and 1B) and statistically significant mean fold increase compared to NBISA01 at all the days except day 3. PKR is the most studied member of the alpha subunit of eukaryotic initiation factor-2α (eIF-2α)-specific kinase subfamily. It is a serine/threonine characterized by two kinase activities: autophosphorylation in response to binding of dsRNA with high affinity and ssRNA with low affinity, and phosphorylation of eIF-2α to impair protein synthesis during virus infection [17]. In addition, PKR has a role in signal transduction control through IκB/NF-κB pathway. NBISA01 haemagglutination showed increase in PKZ transcript levels by day 3 (18.46 ± 0.79) (Fig. 1A). RPC/NB-04-085-1 haemagglutinations did not show specific amplification for PKZ mRNA (data not shown). The negative control erythrocytes kept in L-15 medium had very minimal induction with a maximum 2.34 ± 1.21 relative fold increase of Mx transcripts by day 5 (Fig. 2). The UV-inactivated NBISA01 and RPC/NB-04-085-1 preparations induced very low transcript levels below the 0 hour control (Figs 3A and 3C), whereas the heat-inactivated preparations of both strains showed slight up-regulation of the transcripts (Figs 3B and 3D). These results indicate that fish erythrocytes are immunologically active and produce key Type I IFN genes, particularly IFN-α, upon detection of virus associated molecular patterns.

### PolyI:C stimulated erythrocytes show minimal induction of Type I IFN system genes

**Figure 4:**
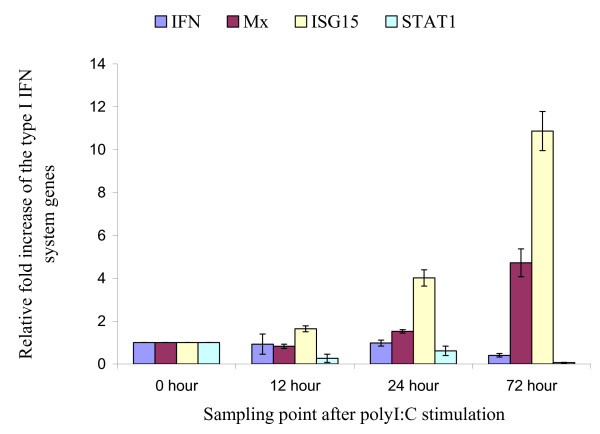
**mRNA levels of key Type I IFN system genes in Atlantic salmon erythrocytes in response to PolyI:C stimulation**. Relative fold increase of the key Type I IFN system genes in response to polyI:C stimulation calibrated to the 18S rRNA housekeeping gene and the 0 hour control (values are average of a triplicate observation ± standard deviation).

PolyI:C is a synthetic double stranded (ds)RNA that simulates viral replication nucleic acid intermediates. PolyI:C stimulation of the TO cell line has been shown to induce the expression of key Type I IFN system genes [19]. PolyI:C was included in this study as a direct positive control for inactivated virus preparations. To determine whether fish erythrocytes respond to polyI:C stimulation, Atlantic salmon erythrocytes were exposed to a large dose of polyI:C and incubated as for the haemagglutination reactions. As shown in Figure 4, there was only minimal induction of the genes investigated except for ISG15 and Mx by 72 hours after stimulation. These results show that, unlike TO cells, Atlantic salmon erythrocytes do not efficiently respond to polyI:C stimulation. However, the response was similar to that of erythrocytes exposed to heat-inactivated ISAV or to L-15 medium alone (negative control assays).

## Discussion

Two models of haemagglutination-infection phenotypes have been proposed to account for the anaemia associated with the clinical disease due to ISAV infection in fish. The first model is that anaemia in the clinical disease is due to uptake by immune cells of fish erythrocytes coated with ISAV [6], and the receptor destroying enzyme (RDE) activity, which is related to the pathogenicity of the virus [13], allows the virus to elute from fish erythrocytes except those of Atlantic salmon [7]. An alternative model is that failure of ISAV to elute from Atlantic salmon erythrocytes favours virus infection of the erythrocytes, which might result in cell death, and this combination is related to the pathogenicity of the virus [11]. Such a haemagglutination-infection phenotype is fundamentally different from haemagglutination by avian and mammalian orthomyxoviruses, and may be indicative of a different pathogenesis for the fish orthomyxovirus.

In the present study we set up haemagglutination assays in L-15 growth medium to compare two phenomena (elution and uptake) of the ISAV-induced haemagglutination of Atlantic salmon erythrocytes between virus strains of differing pathogenicities. We found remarkable differences in virus replication and quality of cytokine response in the fish erythrocytes. Real-time quantitative RT-PCR was used to assess the viral RNA levels (i.e., both vRNA and viral mRNA) in the haemagglutination reaction samples. Only the Ct values for the NBISA01 haemagglutinations showed any decrease from the 0 hour to day 5. This decrease was evident even when oligodT primers were used for cDNA synthesis, confirming that there was *de novo *synthesis of virus genes in the erythrocytes. However, there are relatively long stretches of adenosine residues in the ISAV target gene that could allow detections of vRNA as well. The RPC/NB-04-085-1 haemagglutinations showed no changes in the Ct values at any sampling time point, indicating that the low pathogenic virus did not replicate in erythrocytes. Moreover, using virus titrations in the TO cell line, it was shown that NBISA01 haemagglutinations resulted in a productive infection. The increase in virus titre between day 0 and day 5 was only 10^0.75 ^TCID_50 _in contrast to the 10-fold increase in the viral transcript levels detected by real-time RT-PCR within the same samples. This may be due to three factors: [1] the lower sensitivity of the virus titration in TO cell line compared to real-time RT-PCR, [2] the fact that real-time RT- PCR also detects non infectious or defective particles which the TCID_50 _does not, and [3] the fact that the virus replication associated with haemagglutination involved only a single cycle of virus replication as multiple haemagglutination events were unlikely. It is interesting to note that avian erythrocytes (which have a dormant nucleus, in contrast to the complete nucleus in fish erythrocytes) also demonstrate virus uptake during haemagglutination by influenza A virus and show *de novo *synthesis of viral proteins but not production of new infectious virus particles [5] whereas mammalian erythrocytes that do not have a nucleus have completely lost the capacity for virus replication during influenza A virus-induced haemagglutination.

In addition to virus replication in haemagglutinations induced by the highly pathogenic NBISA01 strain, we found that there was also a higher relative fold increase of IFN-α transcripts than with the less pathogenic RPC/NB 04-085-1 strain which did not replicate in erythrocytes. The induction of the IFN-α gene closely followed the increase of NBISA01 transcripts in that by day 1 the viral transcripts started to increase simultaneously with the first peak of IFN-α transcripts. The NBISA01 haemagglutinations showed a pattern of fold increase with a peak of IFN-α and Mx transcripts for a shorter period of time. This pattern of induction is not continuous like the inductions in TO cells infected with ISAV [19]. This may be due to differences between the cell cycle of TO cells (which actively multiply) and erythrocytes (which do not multiply) in combination with the single cycle of virus replication that probably occurs during haemagglutination in contrast to multiple cycles of infection possible in TO cells. For the NBISA01 haemagglutinations in the present study, the level of IFN-α appeared to have a transient biphasic peak at 1 and 3 days post-haemagglutination.

Both the UV- and heat-inactivated preparations of both NBISA01 and RPC/NB-04-085-1 viruses and the L-15 medium assay (negative control) showed no haemagglutination. The UV-inactivated viruses also showed no induction of type I IFN system genes whereas the heat-inactivated viruses and the L-15 medium assay showed induction of the type I IFN system genes by day 5 similar to those due to polyI:C stimulation by day 3. The absence of haemagglutination in the UV-inactivated viruses was unexpected since the inactivation was directed towards the viral genome and not the surface glycoproteins required for haemagglutination. One possible explanation is that the UV lamp generated sufficient heat over the 18 hours of exposure to contribute to the denaturation of the virus surface glycoproteins. In contrast, the heat inactivation alone had no effect on the viral ssRNA genome but probably even disrupted the structural viral proteins so that the virus RNA was exposed and easily detected by the erythrocyte viral pattern recognition receptors so as to induce the observed minimal induction of the Type I IFN system genes. Alternatively, these minimal responses were non-specific since they were also seen with L-15 medium alone (negative control assays).

STAT-1 expression has been studied in other fish species including rainbow trout [25] but this is the first study to investigate STAT1 expression in Atlantic salmon. It appears that induction of STAT1 is not as highly responsive to IFN induced by virus infection as the other component genes of the Type I IFN system in that the fold increase was low compared to the other genes studied. This could be related to the multifunctional role of this transcription factor.

The NBISA01 haemagglutination showed a moderate relative fold increase of PKZ transcripts. PKR gene has been characterized in rainbow trout [26], and crucian carp [27]. PKR is one of the antiviral proteins of the IFN system [17]. For Atlantic salmon, only the sequence of a Z-DNA binding eIF-2α kinase is available in the GeneBank database. In crucian carp cells, PKR mRNA has been shown to be up-regulated in response to either IFN protein treatment or virus infection. Moreover, in rainbow trout PKR has been shown to be activated to phosphorylate eIF-2α in response to polyI:C stimulation and virus infection. It is very interesting that Atlantic salmon erythrocytes showed expression of PKZ gene, albeit moderate, in response to haemagglutination with a pathogenic ISAV strain.

In the present work the RT quantitative PCR data showed varying levels of induction of key Type I IFN system genes IFN-α, Mx, ISG15, STAT1, and PKZ upon haemagglutination of erythrocytes by the highly pathogenic NBISA01 virus. This virus induced significantly high relative fold increase in IFN-α transcripts compared to the RPC/NB-04-085-1 virus although both viruses had similar levels of induction of Mx, ISG15 and STAT1. The slight Type I IFN system response with RPC/NB-04-085-1 haemagglutinations which involve only virus adsorption but no endocytosis [11] and no replication is an interesting observation. Various viral pathogen recognition receptors are involved in the detection of viral pathogen associated molecular patterns such as dsRNA, ssRNA, DNA, and viral glycoproteins like haemagglutinin proteins. In the case of human cytomegalovirus [28], herpes simplex [29] and human immunodeficiency virus [30], peripheral mononuclear cells have been shown to induce Type I IFN independent of virus replication, purely by the viral glycoproteins. Thus the low level Type I IFN system gene induction detected in the present study for the low pathogenic RPC/NB-04-085-1 virus could possibly be associated with the detection of the viral HE protein during haemagglutination.

PolyI:C is a synthetic dsRNA that is detected either by the RNA helicases or the Toll-like receptor 3 (TLR3) to activate the transcription of type I IFN system genes (reviewed in [16]). This has been shown in the macrophage-like Atlantic salmon TO cell line [19]. Stimulation of erythrocytes with polyI:C did not, however, result in induction of Type I IFN genes even with a polyI:C dose 10 times that used elsewhere [19]. It was previously reported that CHSE-214 cells incubated with polyI:C show no expression of Mx [31], probably because of inefficient response to polyI:C stimulation. In the present study, NBISA01 endocytosis and replication in Atlantic salmon erythrocytes resulted in up-regulation of the IFN-α gene possibly by detection of the viral molecular patterns by the erythrocytes. Thus the minimal induction of the Type I IFN genes in fish erythrocytes by polyI:C could be due to inefficient membrane transport activity of erythrocytes.

## Conclusion

In conclusion, we report here that ISAV-induced haemagglutination by a pathogenic virus strain results in virus uptake and productive infection of Atlantic salmon erythrocytes accompanied by significant induction of IFN-α. This study also highlights the critical role of ISAV strain variation in the initial stages of the virus-cell interaction during haemagglutination, and possibly in the pathogenesis of ISA. Moreover, the study shows for the first time that fish erythrocytes immunologically respond to ISAV infection.

## Methods

### Viruses

Two ISAV isolates of differing genotypes and pathogenicities were used. NBISA01 is a highly pathogenic strain belonging to the North American genotype, whereas RPC/NB 04-085-1 is a low pathogenic strain of the European genotype found in eastern Canada and its HE protein places it in a unique, highly polymorphic region (HPR) group [32]. The two isolates have variations in the amino acid sequence of the HPR region with amino acid deletions of 13 and 17 amino acids for RPC/NB-04-085-1 and NBISA01, respectively [33]. The viruses were propagated in the TO cell line [18] and the cell lysates were titrated on TO cell monolayers as previously described [34] prior to use in the subsequent studies.

### Virus inactivation

The viruses were inactivated by using either UV light or heat treatment. UV inactivation of ISAV was carried out with a germicidal UV lamp (G30T8 with 30 Watt and 36 inch length, and a UV intensity of 125 μW/cm^2 ^at 1 meter from the lamp) suspended in a biological safety cabinet (Class II A/B3 BSC, Thermo Forma) following the procedure reported by Oye and Rimstad [35], with minor modifications. Briefly, 20.0 mls of virus suspension in a 4-well cell culture plate were placed 10 cm from the UV lamp. The plate was left open under UV-exposure for 18 hours. Heat inactivation of ISAV was performed by incubating 1.0 ml of the virus suspension in a 1.5-ml microfuge tube at 56°C for 5 minutes. Complete inactivation of virus by both methods was confirmed by titration in TO cell monolayers [34] before use in the haemagglutination reactions.

### Haemagglutination assays

Atlantic salmon erythrocytes were collected from specific pathogen free 100 g-Atlantic salmon using EDTA-coated Vacutainer^® ^tubes. In preliminary experiments, the washed erythrocytes suspended in phosphate buffered saline (PBS) were not viable beyond 48 hours. Therefore, common fish cell line growth media, Leibovitz's L-15 (Invitrogen) and Hanks minimum essential medium (BioWhittaker) (HMEM), were tested to identify one that better maintained erythrocyte viability. Using the Trypan blue dye exclusion test, we found that erythrocytes resuspended in L-15 growth medium had lower cell deaths, and those surviving maintained a normal shape in contrast to erythrocytes in HMEM growth medium which were shrunken. In subsequent experiments, the erythrocytes were washed and then resuspended in L-15 medium supplemented with 10% foetal bovine serum, 2 mM L-glutamine, 100 IU/ml penicillin G, 100 μg ml^-1 ^streptomycin, and 0.25 μg ml^-1 ^amphotericin B. For determining the haemagglutination (HA) units of the stock virus preparations, haemagglutination reactions were set up using 50 μl of two-fold dilutions of the two virus isolates and 50 μl of 1% erythrocytes [36]. The haemagglutination was set using four wells for each virus dilution; 1 HA unit was defined as the highest virus dilution that induced haemagglutination in four wells within 1 hr at room temperature. Subsequent haemagglutination reactions used 1 HA unit in 50 μl of virus preparation 50 μl of 1% erythrocytes in L-15 growth medium. The sealed plates were kept at room temperature for one hour, and then transferred to 16°C for the extended incubation until sampled.

### PolyI:C stimulation of Atlantic salmon erythrocytes

Washed Atlantic salmon erythrocytes were resuspended in L-15 medium consisting of 10% FBS, 2 mM L-glutamine, 100 IU ml-1 penicillin G, 100 μg ml-1 streptomycin, and 0.25 μg ml-1 amphotericin B, and polyinosinic:polycytidylic acid (polyI:C) (Amersham Biosciences) at a final concentration of 30 μg ml-1. One hundred microliters of 1% erythrocyte suspension was added to each well of the haemagglutination plate and incubated at 16°C. The preparations were sampled after 12, 24, and 72 hours.

### Detection of cytokine induction and virus replication using real-time RT-PCR with SYBR Green chemistry

Total RNA from the haemagglutination samples was extracted from 375 μl of homogeneous erythrocyte suspensions using 1.25 ml of TRIZOL Reagent (Invitrogen). RNA extraction was performed from two separate samples at each sampling point, which were then pooled before DNase treatment using the DNase treatment kit (Roche) prior to RT-PCR amplification.

For quantification of the Type I IFN system genes and viral RNA, first strand cDNA synthesis was done using the Transcriptor reverse transcriptase first strand cDNA synthesis kit (Roche). The cDNA synthesis used 125 ng of total RNA in a master mix consisting of 4 μl of 5x RT reaction buffer, 2 μl of dNTP mix (200 μM), 2 μl of random hexamer (600 μM) or oligodT (0.8 μg/μl), 0.5 μl RNase inhibitor (40 U/μl), 0.5 μl of Transcriptor reverse transcriptase (20 U/μl), and nuclease free water adjusted to a final volume for 20 μl. The RT step was programmed at 25°C for 10 minutes followed by 55°C for 30 minutes and a final enzyme denaturation for 5 minutes at 85°C. Real-time PCR used first strand cDNA template with LightCycler FastStart DNA Master SYBR Green I (Roche) in the LightCycler (LC) 1.2 (Roche). The PCR primer pairs used are listed in Table 2; those for 18S rRNA, IFN-α, Mx, and ISG-15 are published [19], and the STAT-1 primer pair was described in Workenhe [37]. The PKZ (a Z-DNA binding orthologue of the mammalian double stranded RNA binding PKR) primer was designed using the coding sequence of Atlantic salmon Z-DNA binding eIF-2α kinase (GenBank Accession # DQ182560). The IFN gene primer set is designed in the common region of the two IFN-α subtypes, α1 and α2 [38]. The 20 μl PCR reaction consisted of 2 μl of undiluted cDNA for all genes except 18S rRNA (which was diluted 1:1000) and 18 μl of the master mix prepared using 0.5 μl of the 10 μM of the forward and reverse primers (a final concentration of 0.25 μM), 2 μl of the LC SYBR Green I DNA Master mix, 1.6 μl of the stock 25 mM MgCl_2 _(a final concentration of 0.003 μM), and 13.4 μl of nuclease free water. The real time PCR programme for amplifying PKZ gene had a master mix consisting of 12.8 μl of water, 2.4 μl of 25 mM MgCl_2 _(a final concentration of 0.004 μM), 0.4 μl of the 10 μM forward and reverse primer, and 2 μl of SYBR Green master mix. The real-time PCR cycling conditions consisted of an initial denaturation at 95°C for 10 minutes to activate the hot-start polymerase, followed by 40 cycles of 95°C for 5 s, 59°C for 10 s (60°C for the PKZ gene), 72°C for 10 s, and detection at 80°C for 2 s. The cycle threshold (Ct) values, the number of cycles run in real-time RT-PCR when the fluorescence in the sample crosses a threshold value (background) and amplification enters a log-linear phase, were analyzed using LightCycler software version 3.5 (Roche). Melting curve analysis with the same software was performed from 70°C to 95°C in 0.1°C/s increments to verify the specificity of the amplicons so as to interpret SYBR Green fluorescence data. For determining amplification efficiency of each primer set (Table 2), standard curves were generated using two-fold dilutions of cDNA run in triplicates for six consecutive dilutions. Each sampling point was run in triplicate and the stability of the 18S rRNA, used as housekeeping gene, was followed. The Ct values of positive amplicons were then analyzed using the Pfaffl method for relative quantification in real-time RT-PCR [39] as previously used elsewhere [19], to get relative fold increase of the Type I IFN genes at each sampling point calibrated to the house keeping gene and normalized with the 0 hour control. To test if the difference in mean relative fold induction between the two virus isolates at each sampling point was statistically significant, data were initially checked for equality of variance using F- test in Microsoft Excel spread sheet. Then the t- Test was used considering the equality/inequality of variance where applicable [40].

**Table 2:** The oligonucleotide primers, amplicon length and amplification efficiency of the real time RT-PCR primers amplifying IFN, Mx, 18S, ISG15 and STAT1 genes

Primer name	Primer sequence	Amplicon length (bp)	Amplification efficiency
As IFN Fwd^1^	TGCAGTATGCAGAGCGTGTG	100	1.83
As IFN Rev^1^	TCTCCTCCCATCTGGTCCAG		
As Mx Fwd	TGCAACCACAGAGGCTTTGAA	77	1.88
As Mx Rev	GGCTTGGTCAGGATGCCTAAT		
As 18S Fwd	TGTGCCGCTAGAGGTGAAATT	60	1.86
As 18S Rev	GCAAATGCTTTCGCTTTCG		
As ISG15 Fwd	CTGAAAAACGAAAAGGGCCA	100	1.83
As ISG15 Rev	GCAGGGACTCCCTCCTTGTT		
As STAT1 Fwd	TGTCTGTTGGCTCAGTTGCG	100	1.82
As STAT1 Rev	GAAATTGATGCTGTGGCGTCT		
As PKZ Fwd	AGATAGCGAAGGCTGTTGGA	101	1.913
As PKZ Rev	TGGTTTGTCTGGTGTTGCAT		

^1^Primer amplifies SasaIFN-α1 and α2.

For quantifying the level of viral RNA, real-time RT-PCR was done using the RNA Amplification Kit SYBR Green I (Roche) and the primer pair designed by Devold *et al*. [41] to amplify 220 bp of the ISAV segment 8, and previously described for real time RT-PCR [42], with minor modifications. Briefly, the 20 μl reaction consisted of 50 ng of total RNA in a master mix prepared using 0.3 μl of the 20 μM of the forward and reverse primers (final concentration of 0.3μM), 4 μl SYBR Green, 0.2 μl LC-RT PCR enzyme mix, 3 μl of the 5x resolution solution, 1.6 μl of the 25 mM stock MgCl_2 _(a final concentration of 0.005 μM), and nuclease free water adjusted to a final volume of 20 μl. The cycling conditions consisted of one cycle of RT at 55°C for 30 min, initial denaturation at 95°C for 30 s followed by 50 cycles of 95°C for 5 s, 59°C for 10 s, 72°C for 10 s, and detection at 80°C for 2 s. The Ct values and melting curve data were analyzed using LightCycler software version 3.5 (Roche). Melting curve analysis was performed from 70°C to 95°C in 0.1°C/s increments to verify the specificity of the amplicons so as to interpret SYBR Green fluorescence data. The amplicons were also run in 1% agarose gel electrophoresis in 1x Tris acetate EDTA buffer (40 mM Tris acetate and 1 mM EDTA) (Fisher Scientific) and stained with ethidium bromide and photographed under 304 nm UV light.

### Detection of virus replication by titration on TO cell monolayers

Total cell lysates of the haemagglutination assays (i.e., total virus) were titrated to determine growth cycles of the virus strains in Atlantic salmon erythrocytes. Virus titration utilized serial 10-fold dilutions of the samples ranging from 10^-1 ^to 10^-8^, inoculated on 48-well cell culture plates containing TO cell monolayers using 4 wells per dilution, from which the median tissue culture infectious dose (TCID_50_) was determined as previously described [33]. Each sampling point was titrated in triplicate to obtain a standard deviation.

## Competing interests

The author(s) declare that they have no competing interests.

## Authors' contributions

STW conducted all the experiments and wrote the manuscript. MJTK helped in designing the experiments and writing the manuscript. GMW and DWW helped in the initial stages of conceiving the study and edited the manuscript. DBG helped in designing the experiments and edited the manuscript. FSBK conceived the study, coordinated the research, and helped in designing the experiments, writing and editing the manuscript.
